# Supplementary figures and images for: A systematic screen identifies Saf5 as a link between splicing and transcription in fission yeast
Source: PLoS Genet. 2024 Jun 4;20(6):e1011316. doi: 10.1371/journal.pgen.1011316 (PMC11178228; doi:10.1371/journal.pgen.1011316)

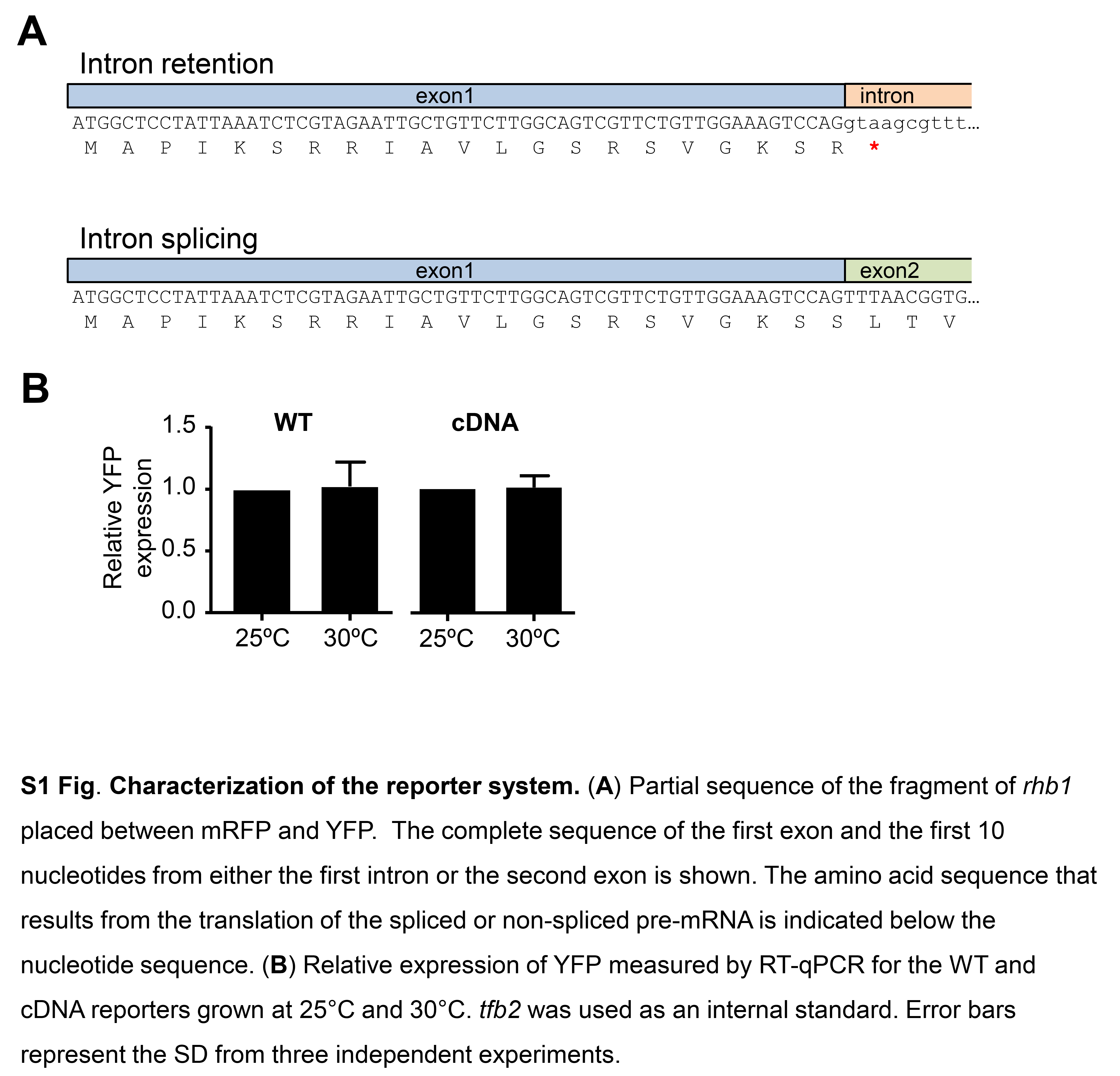

Supplement: S1 Fig — (TIF) [file pgen.1011316.s006.tif]

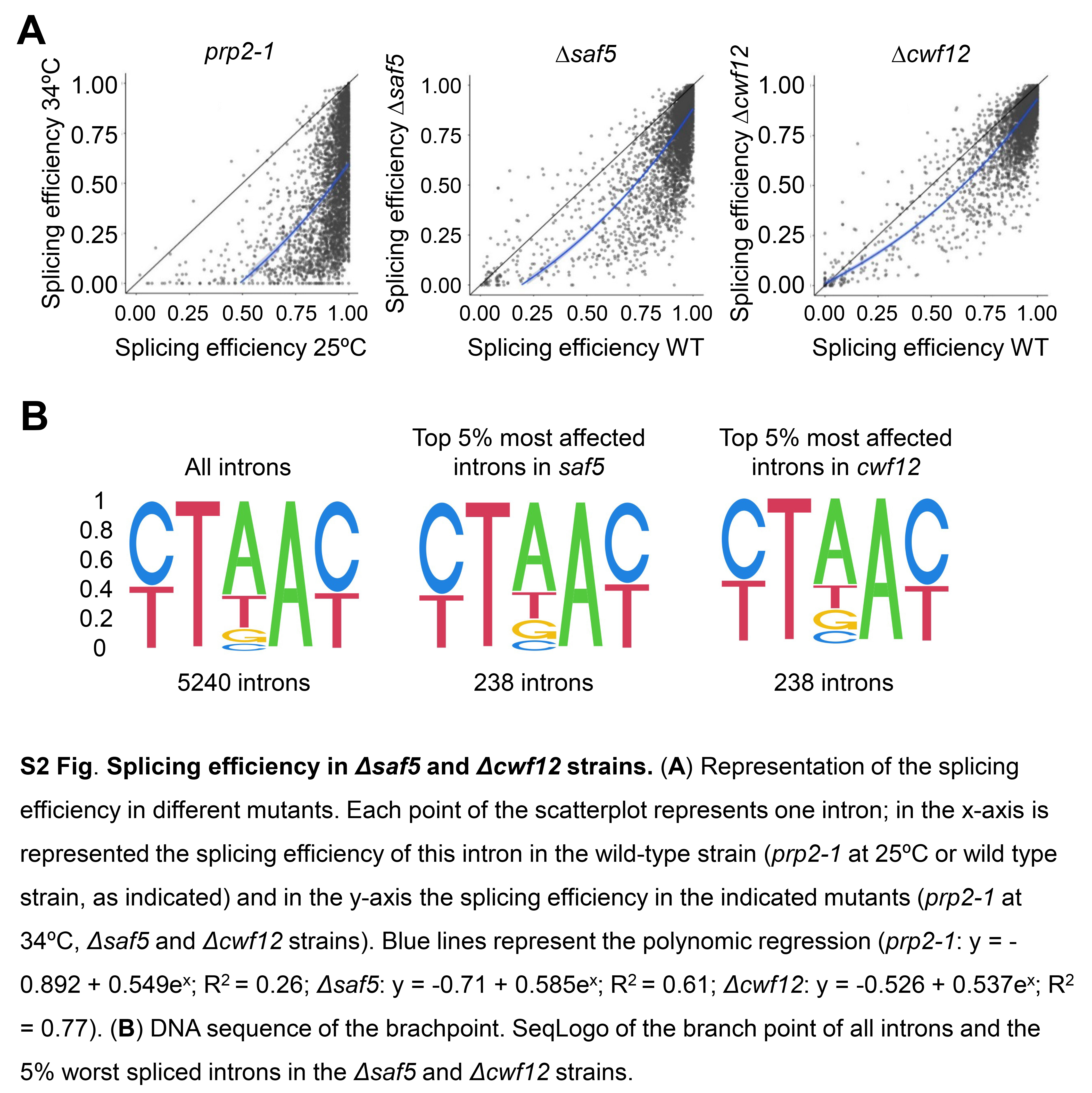

Supplement: S2 Fig — (TIF) [file pgen.1011316.s007.tif]

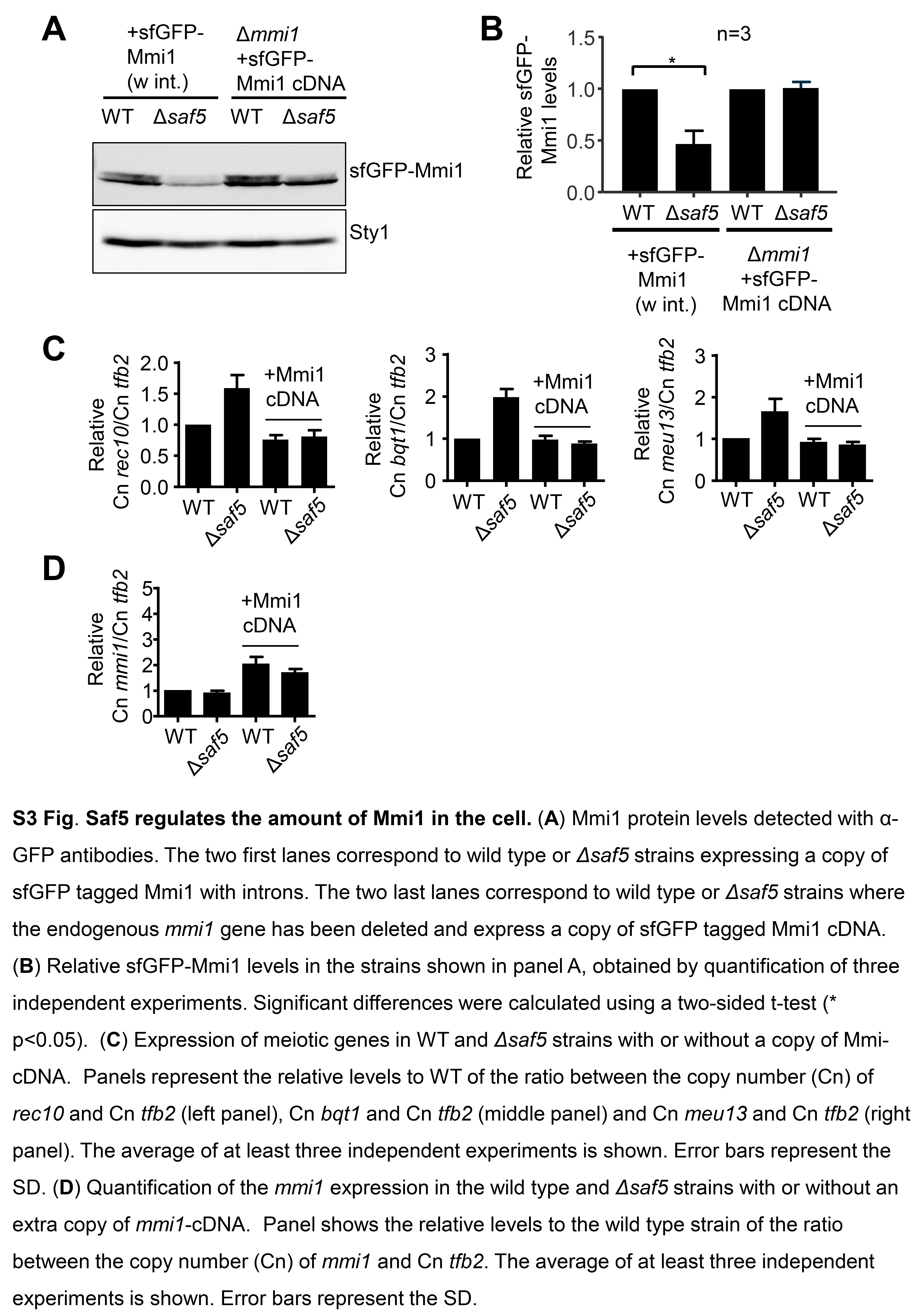

Supplement: S3 Fig — (TIF) [file pgen.1011316.s008.tif]

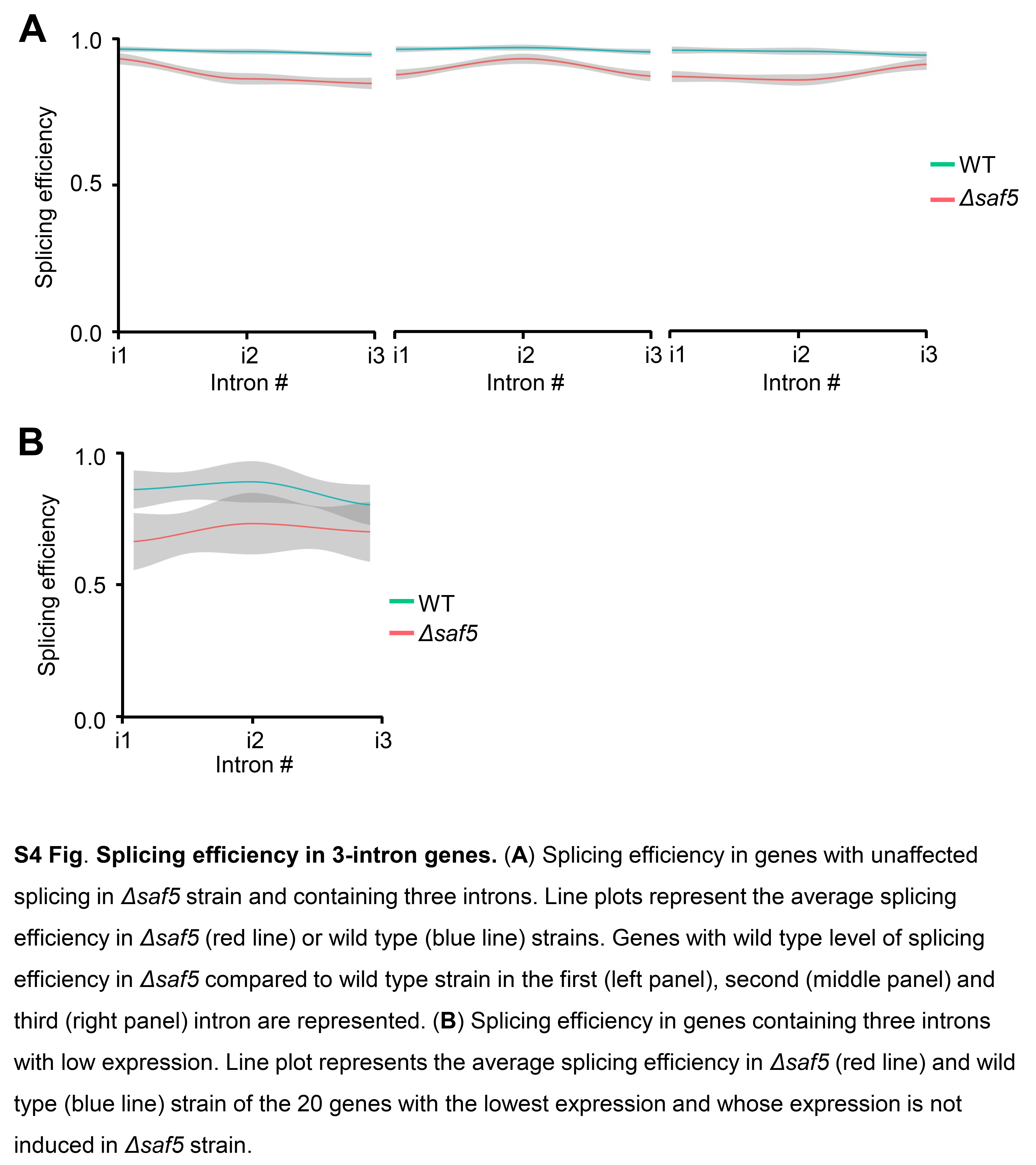

Supplement: S4 Fig — (TIF) [file pgen.1011316.s009.tif]

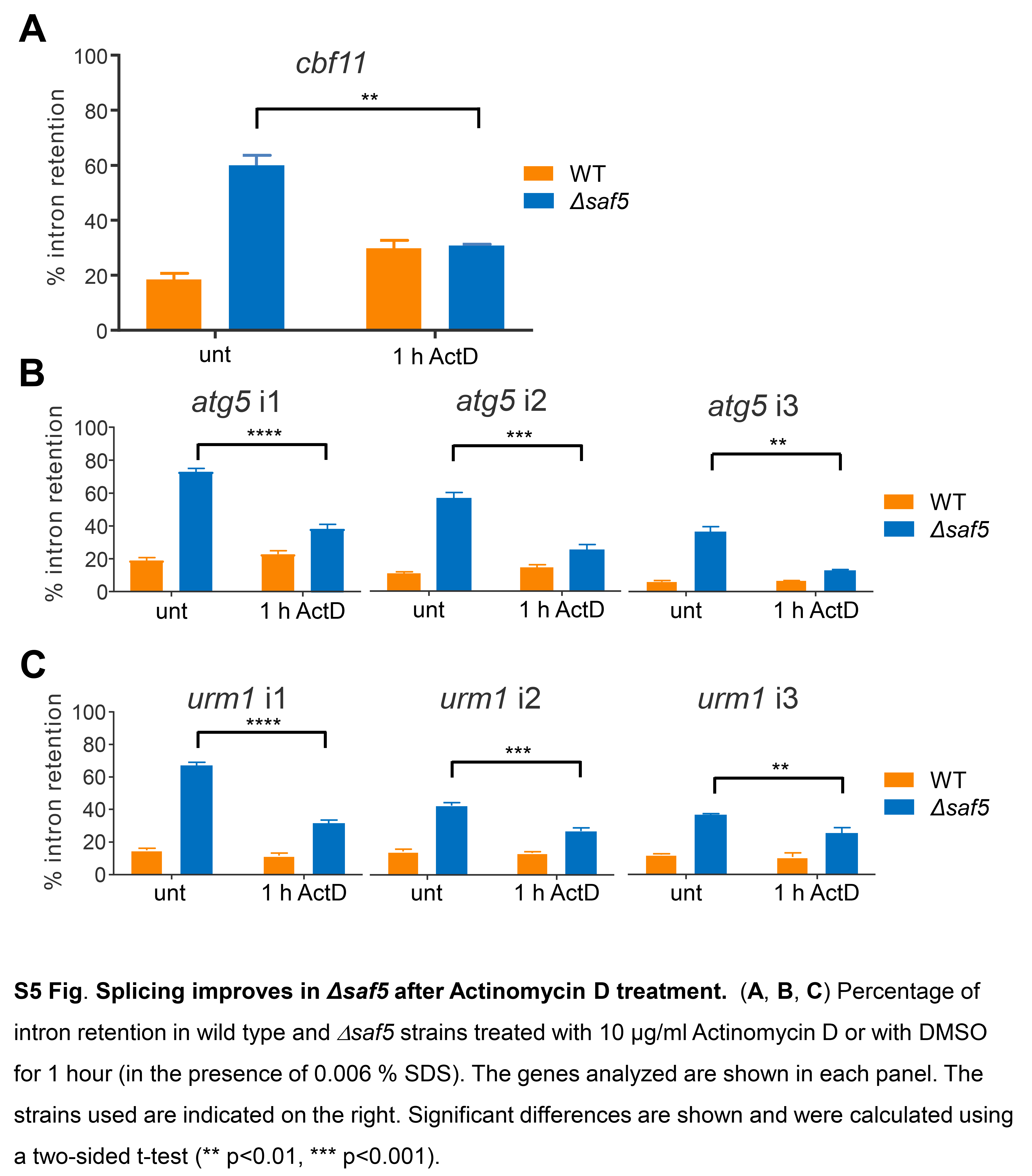

Supplement: S5 Fig — (TIF) [file pgen.1011316.s010.tif]
