# Supplementary material for: A systematic screen identifies Saf5 as a link between splicing and transcription in fission yeast
Source: PLoS Genet. 2024 Jun 4;20(6):e1011316. doi: 10.1371/journal.pgen.1011316 (PMC11178228; doi:10.1371/journal.pgen.1011316)
Supplement: Attachment — Submitted filename: Response to Reviewers.pdf [file pgen.1011316.s012.pdf]

Reviewer #1: Borao et al. In the submitted manuscript, the authors screen the S. pombe deletion library using fluorescence-based reporters to identify new genes with links to splicing efficiency. They identify the Cwf12 and Saf5 genes for follow up work, noting that Saf5 but not Cwf12 mutants have defects in splicing of endogenous genes transcriptome wide. Noting that genes most sensitive to Saf5 deletion correlate with high expression and impaired splicing in multiple introns of the same gene, they hypothesize that splicing defects linked to Saf5 deletion may be related to transcription elongation. This is tested using a P-TEFb/Cdk9 mutant allele that indeed suppresses splicing defects in the Saf5 deletion background. The authors conclude that Saf5 likely has functions related to splicing in a co-transcriptional context.

The manuscript is very interesting and the use of the fluorescence-based screen across an entire deletion library is impressive and appealing. The validation of the reporters in the context of the prp2-1 allele is reassuring.

We would like to thank the encouraging comments from the reviewer. As she/he will observe, we have tried to follow all her/his recommendations, which have noticeably improved our manuscript.

Comments:

- The link between transcription elongation and suppression of Saf5 defects seems underdeveloped. The rescue in the context of a Cdk9 mutant allele is very appealing, but since Ser2 phosphorylation is linked to both transcription elongation rates and recruitment of splicing factors, it seems premature to conclude that the effects they see must be due to changes in elongation rates.
- Related to this, the authors should articulate what they mean by "transcription rate" when they refer to highly transcribed genes; they have only tested for effects on transcription elongation rates, but highly transcribed genes will also be intimately linked to transcription initiation events/rates. Ideally the authors would validate the hypothesis linking Saf5 to transcription elongation using another method that does not rely on Ser2 phosphorylation, such as a drug (Actinomycin D?) or a mutant of RNA polymerase that has slower elongation rates. Alternatively, to at least rule out an effect due to initiation rates, an intron containing reporter with an inducible promoter could be used, to show that low versus high transcription initiation rates are not linked to Saf5 associated changes in splicing efficiency.

Following the reviewer recommendation, we have tested the effect of Actinomycin D on splicing in WT cells and in *Δsaf5* cells (there are no described RNA polymerase mutants in fission yeast with slower elongation rate). As can be observed, splicing efficiency is improved when *Δsaf5* cells are treated with Actinomycin D, confirming our previous results with the Cdk9 mutant allele (see new Supporting Figure 5).

We have also tested the effect of Ser5 phosphorylation using alleles that abolish or diminish Ser5 phosphorylation (*lsk1-as*) in the presence of bulky ATP (same inhibitor as the one used to inhibit Cdk9_as. As the reviewer can observe, when Ser5 phosphorylation was abolished, there was no effect on

splicing (we are showing here the effect on the *cbf11* single intron, but have obtained the same results with the other introns that we have tested in the manuscript)

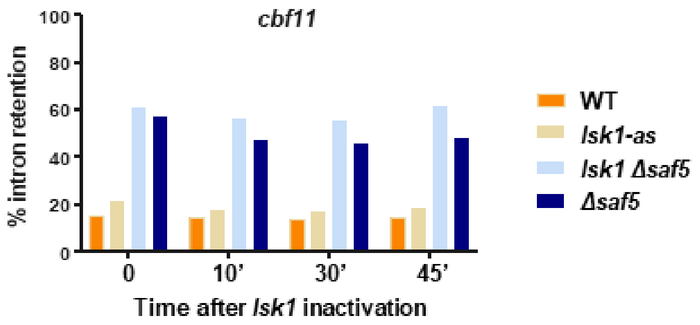

- I would like to see the equivalent data in Figure 4F for multi-intron genes that are not highly expressed and whose expression was not affected by deletion of Saf5. I think this is an important control that would help substantiate the main hypothesis.

We have now added a supplementary figure 4B showing the splicing efficiency of three intron containing genes with low expression levels (and whose transcription is not affected in the absence of Saf5). As it can be seen, splicing efficiency of low expressed genes remain in acceptable levels (compare with figure 4F):

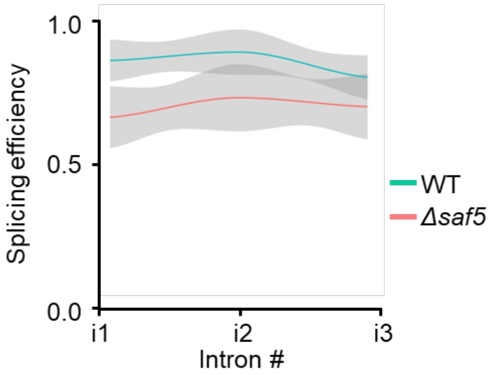

- The Discussion is quite brief. It would be helpful to bring up other work linking transcription elongation rates & Rbp1 CTD phosphorylation to efficiency of splicing as this literature is quite extensive. Further discussion of links to the SMN complex, its function in snRNP assembly and conservation of this process in budding yeast, fission yeast and humans would also be appropriate.

We have extended the Discussion of our manuscript, following the 2 topics suggested by the reviewer in 2 different paragraphs:

> *Saf5 is conserved from yeast (LOT5 in S. cerevisiae) to metazoans and the human ICLN gene (CLNS1A) complements the Δsaf5 slow-growth phenotype [32]. ICLN, together with WD45 and the PRMT5 methyltransferase forms a complex termed methylosome that acts together with the SMN complex during the early steps of snRNP biogenesis [38]. The core spliceosome consists of five snRNPs and the*

biogenesis of these snRNPs is an ordered multistep process where ICLN acts as assembly chaperone assisting in the recruitment and the formation of the Sm core protein complex on the snRNA [39]. Although these functions have not yet been rigorously confirmed for Saf5 in S. pombe, the functional complementation by the human homolog points towards a conserved role.

Research spanning several decades has provided substantial evidence indicating that a significant portion of splicing occurs co-transcriptionally. Transcriptional dynamics can influence the selection of splice sites within pre-mRNA, thereby leading to alternative splicing events and the generation of diverse mRNA isoforms [40-43]. The functional interplay between transcription and splicing is proposed to be governed by spatial and kinetic mechanisms [44]. Central to both mechanisms is the carboxy-terminal domain (CTD) of RNA Polymerase II (Pol II), whose phosphorylation status regulates transcription elongation rates and facilitates physical interactions with splicing factors such as Prp40, thereby facilitating association with the U1 complex [45]. The kinetic model posits that differential Pol II elongation rates may dictate the availability of splice sites [42, 44]. The data presented herein unequivocally establish a correlation between highly transcribed genes and the indispensable role of Saf5 in ensuring efficient splicing. Given its functional homology to ICLN, it is tempting to speculate that the absence of Saf5 creates a bottleneck in the biogenesis of functional snRNPs. The functional coupling of rapid transcription rates with splicing may place a heightened demand on snRNPs, with highly transcribed genes relying particularly on Saf5 for adequate snRNP supply. In scenarios where snRNP levels are insufficient, splicing and transcription may become uncoupled and fail to match the pace of transcript production. This potentially elucidates why the splicing of highly transcribed genes exhibits heightened dependence on Saf5. Future investigations will be required to validate this hypothesis and deepen our understanding of the intricate relationship between transcription, splicing, and snRNP biogenesis.

Minor issues:

There are some minor English languish issues in the manuscript which would benefit from editorial revision, including but not limited to:

- The term “life construct” is not commonplace; perhaps “integrated fluorescence-based in vivo splicing reporter construct”, or some other version of this?

We have changed “life construct” with “integrated fluorescence-based *in vivo* splicing reporter construct”

- Abstract: “weakest splicing rate”; the term “rate” is typically linked to a quantitative measure of enzyme kinetics (i.e. k) which is not tested here.

We have replaced that sentence with “we have crossed the reporter **showing more intron retention** with the *Schizosaccharomyces pombe* knock out collection”

- Some description of the Nineteen Complex would be helpful and appropriate. There are previously established links (i.e. Syf1), between the NTC complex and transcriptional elongation, and so the authors should contextualize their results in light of these. Both Cwf12 and Saf5 are indicated in the abstract as being related to the NTC (line 25) but in the text (line 171) only a link from Cwf12 to NTC is provided. Clarification needed.

The reviewer is right and we would like to apologize, but there was a confusion between Saf1 (which is truly related to NTC) and Saf5. This has now been corrected and we have changed the text accordingly. Since we have not done anything else on Saf1 in the manuscript, we have only minimally extended the Discussion with the role of NTC in splicing regulation: “*Cwf12 is a core component of the NTC complex whose founding member is Prp19. Although not being part of the core spliceosome, the NTC complex is associated with the splicing machinery and is required for the two catalytical splicing reactions [37]. Saf1, another candidate identified in the screen, has also been shown to interact with the NTC complex [38].*”

- Initial work (Figure 1) is performed with integrated versions of the reporters, but were plasmid versions used for Figure 2A (line 132; “transformed/transformants”)? This should be clarified.

Everything is done with plasmids, which were integrated in different fission yeast strains. We have changed that sentence to: “*Cultures with the different integrative plasmids were grown at the permissive temperature (25ºC) or subjected to a 5-hour incubation at the semi-restrictive temperature (30ºC).*”

- The difference between how candidates were identified in 2C and how candidates were confirmed in Figure 2D is not clear. Is the assay in 2D the same as what was done in the initial screen in 2C?

2C is a volcano plot of the screening of the collection of mutants (3400 different strains) assayed on 96-well plates, while 2D corresponds to individual cultures of 5 potential candidates that were grown in individual flasks. We have changed the text in the figure legend to point the difference. In fact, fission yeast cells grow much better on individual flasks than in 96-well plates (due to oxygenation problems), which can induce small differences in the read-out. But screening 3400 different strains forces to grow cells in 96-well plates.

Reviewer #2: Borao, Vega and colleagues report the results of a screen in S. pombe for genes important for efficient splicing. Using bichromatic reporters, they first validate their experimental set up by showing that in a Prp2 ts mutant strain, reporters harboring mutations in the 5' splice site (or in the 5'SS and the branchpoint) show decreased splicing under conditions of partial inactivation of Prp2. A pilot screen using 41 ko strains of genes encoding factors involved in RNA metabolism identified cwf12, saf5 and saf1 as displaying a splicing defect when studied using the 5'SS or double mutant reporters. A genome-wide screen confirms the cwf12 and saf5 hits and reveals over 30 additional gene hits. The authors then focus on cwf12 and saf5. RNA-seq analyses do not detect splicing defects in the cwf12 ko strain but substantial defects in the saf5 ko strain, not associated with detectable differences in splice site sequences, A/T content or intron size. saf5 deletion had a profound effect on gene expression, particularly enhancing mRNA levels of over 200 genes, including genes normally expressed only in meiosis. Following this lead, the authors study splicing of introns in the mmi1 gene, which encodes a protein that targets mRNAs encoding meiotic genes for degradation during growth and suggest that cumulative splicing defects in each of these introns explain the effects on overall expression of the gene, with consequences on the upregulation of meiotic gene targets. They find that the genes containing the most affected introns by saf5 ko are generally more expressed. Finally, the authors tested whether decreasing RNA pol II elongation rates by Cdk9 inactivation affected the effects of the absence of saf5 and conclude that slowing down transcription relieves the inhibition of intron splicing induced by saf5 deletion.

These are interesting results that connect saf5 with splicing efficiency and transcription and describe coordinated effects on multiple introns of the same primary transcript. As the mechanisms of coupling between transcription and splicing remain an important open question, the results by Borao, Vega et al provide new information that can trigger further work on this topic.

We would like to thank the encouraging comments from the reviewer. As she/he will observe, we have tried to follow all her/his recommendations which, as we believe, have noticeably improved the manuscript.

In my opinion the following revisions could help to improve the manuscript:

1) It is not straightforward to interpret, from the scheme in Figure 1A, why the unspliced transcript should not produce YFP.

The unspliced transcript includes the first intron from *rhb1*, that is 58 nucleotides long; all the mRNAs that include this intron have a premature STOP codon which is encoded by that intron sequence.

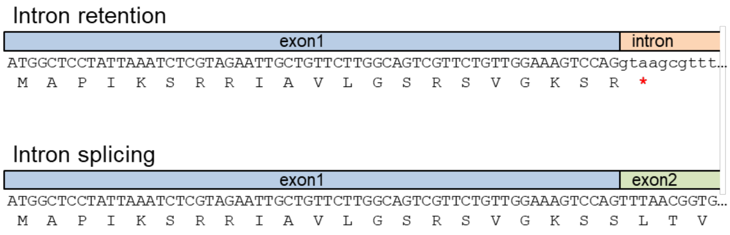

We have included now a full description of this in the supplementary info (Supplementary Figure 1) including the partial sequence of the intron. We have modified Figure 1A to indicate that there is a STOP codon in the intron.

2) Figure 1C: the behavior of the cDNA construct serves as the reference for the YFP/RFP values of the other constructs in each of the two conditions, but is the expression of the cDNA identical under restrictive and under permissive conditions?

We have now quantified the expression of the cDNA and the WT reporters by Q-PCR. The reviewer can observe that there are no significant differences between the 2 reporters or at the different temperatures.

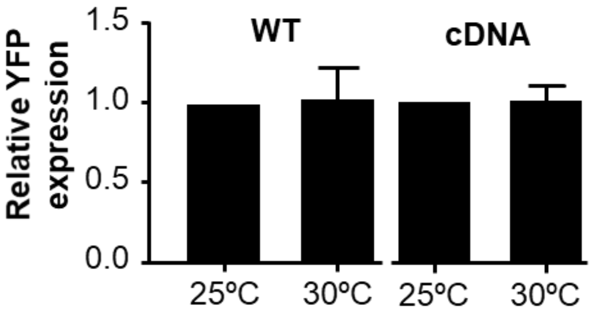

We have included this quantification in the Supplementary Figure 1.

3) I would favor showing the RNA analysis of Supporting Figure 1 in Figure 2 because it convincingly confirms that the bichromatic reporter read out is linked to splicing defects.

Following the reviewer recommendation, we have moved this information to main Figure 2.

4) Figure 2B: are the differences between wild type and mutant strains statistically significant for the WT reporter (0.7-0.9 ratios)? Deltasaf1 is not shown as statistically significant for the dmut reporter in Figure 2B but it is statistically significant in Figure 2D. And again not statistically significant in Figure 2C.

No, in Figure 2B, although the readout (YFP/RFP ratio) in *Δsaf5* and *Δcwf12* is diminished when compared to the wild type strain, the differences are not statistically significant.

Also, when we screened the whole collection of mutants (3400 strains) with the dmut reporter, while *Δsaf5* and *Δcwf12* resulted statistically significant, *Δsaf1* was not (in Figure 2C, *Δsaf1* is the light blue spot that is below the threshold of the p value).

However, in Figure 2D, the *Δsaf1* value is statistically significant ($p < 0.05$), as the reviewer points out. This happened because in the Figure 2D we grew the cultures in individual flasks, while in the rest of the experiments shown in this figure, all the strains were grown in 96-well plates. This, although it may be

trivial, reflects how well the different cultures were grown: when yeasts are grown in 96-well plates they are not under optimal growth conditions and oxygenation is not as high as when they are grown in flasks. But when dealing with an entire collection of mutants (or a sub-collection as in Figure 2AB), it forces to grow yeasts in 96-well plates.

5) Lines 161-163: SPAC1705.02 is mentioned both as one of the four hits previously involved in the regulation of splicing and as one of the remaining 33 hits not related to splicing.

Thanks, we have corrected this typo. We meant the “37 candidates shown in Table 2”.

6) The result with mpn1 is interesting and Ref. 30 reports a function for mpn1 in U6 snRNA processing: it would be good to discuss possible mechanisms potentially explaining the hit, particularly in the light of the functions of Cwf12 in connection with U6 snRNA function.

Thanks for the suggestion. The reviewer is right: there could be a connection between both candidates, since both are related to U6 function; however, given that we have not included more in deep work with Mnp1 and that we have extended the Discussion section on the connection between splicing and transcription rate (as suggested by the 3 reviewers), we believe that adding more discussion on the role of Mpn1 would not help with the linearity of the manuscript.

7) Figures 3C/3D: it is not very clear what is represented in these graphs. Do Top 5% and Bottom 5% indicate introns with the highest variation compared to wild type? In that case “Splicing efficiency” in the X axis should indicate that this is a relative value. More generally, how do the 5% Top and 5% Bottom efficiently spliced introns in the WT compared with the 5% Top and Bottom efficiently spliced introns in the two mutant strains? And how do the Intron size and A/T content compare for these two categories in the three different strains?

The Top 5% and Bottom 5% introns from Figure 3C and 3D were selected comparing splicing efficiency in *Δsaf5* or *Δcwf12* strains, relative to the wild-type strain. We have now changed the label of x-axis to: “Relative splicing efficiency”. The idea behind these two figures is to determine if there was any difference in size or nucleotide composition between the best and the worst introns (in terms of splicing) in these two strains.

Regarding the second question of the reviewer, this is what we have obtained when we analyzed the best 5% or the worst 5% introns in the wild type strain:

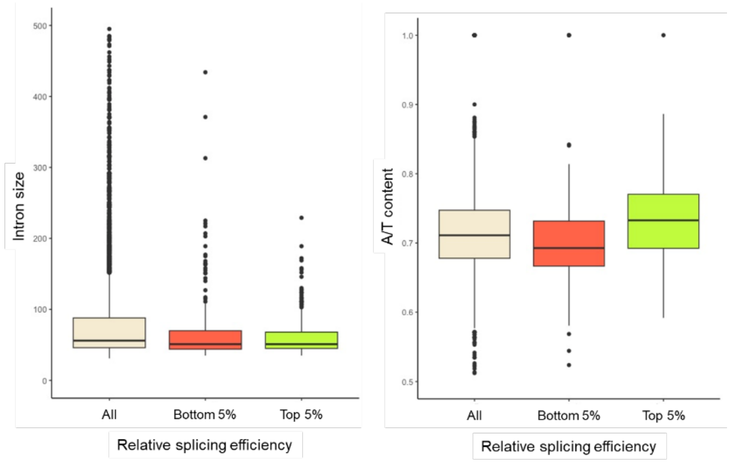

As the reviewer can observe, this graphs are very similar to the one in Figure 3CD, which means that in WT pombe there is no preference for intron size or A/T composition in regular splicing in the WT strain (like in *Δsaf5* or *Δcwf12* strains). However, we have shown in the past (Hummer et al, 2021) that a prp2-1 mutant has some bias in intron size, where introns that are highly dependent on Prp2 function have a tendency to be slightly smaller.

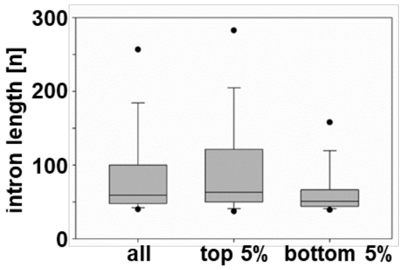

8) I find the result of Supporting Figure 3B very relevant for the authors' case and I would encourage them to include it in the main Figures. Why are two bands detected for sfGFP-Mmi1 in the western blot of Supporting Figure 3A?

Initially, we were very enthusiastic regarding the role that *mmi1* missregulation could have in the *Δsaf5* cells: we though that the phenotype observed in this strain could be explained because these cells have less than 50% of the Mmi1 present in the WT. However, we could not revert the phenotype associated to *Δsaf5* when we replaced the endogenous *saf5* with a *saf5* without introns, indicating that the phenotype associated to *Δsaf5* was of multigenic origin. Since now it is a secondary observation derived from our work, we believe that it is better suited if it remains in the Supplementary information.

Regarding the 2 bands detected in the WB, be believe that are caused by phosphorylation of Mmi1, since they collapse when treated with alkaline phosphatase, although we decided to not analyze them in further detail. Here there is a WB of TCA protein extracts treated or not with Alkaline phosphatase with or without phosphatase inhibitors (ß-glycerophosphate, sodium fluoride and orthovanadate). Although, the upper band (presumably a phosphorylated form) is not very prominent, it clearly disappears upon treatment with phosphatase and the shift is abolished in the presence of inhibitors, suggesting the two bands indeed correspond to phosphorylated and non-phosphorylated forms.

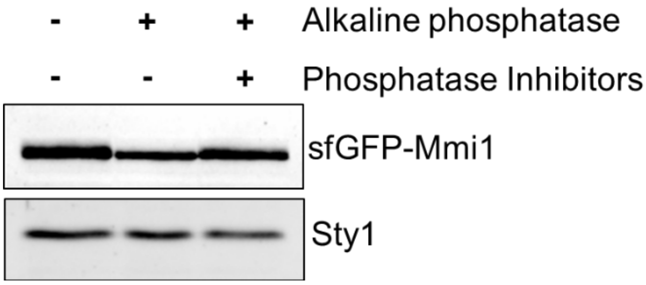

9) The result of Figure 4E makes a point but it may be more compelling to plot splicing efficiency in deltasaf5 vs FPKMs (or use a heat map of splicing efficiencies to paint the dots of the volcano plot in Figure 4C).

We have tried many different representation options to show the relationship between expression and splicing in *Δsaf5*; including the one suggested by the reviewer (splicing efficiency vs FPKMs):

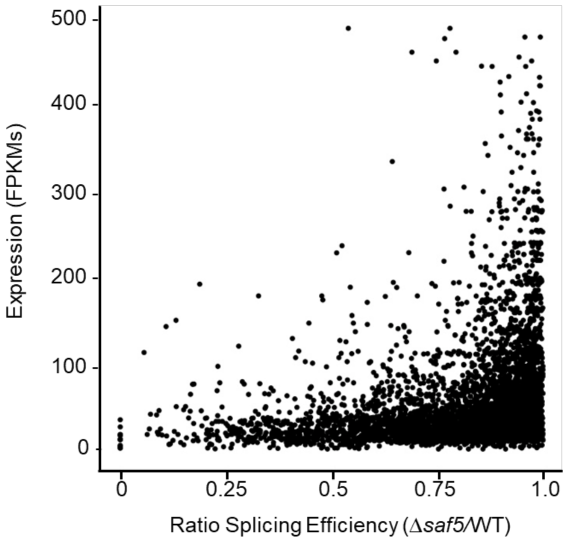

Regarding the volcano plot in Figure 4C it represents the differential expression of *Δsaf5* cells compared to WT and does not correlate with absolute expression levels.

However, we found that the representation as it is shown in Figure 4E is the clearest to show that the higher expressed genes are the ones with the worst splicing efficiency, showing differences between the two groups which are statistically significant ($p<0.001$).

10) The result of Figure 4F is clear but it would be good to see whether the converse if also true, i.e. if, in genes containing multiple introns (e.g. 3) that do not display defects in splicing of the first intron, the splicing of subsequent introns is also not affected by saf5 depletion.

Yes, the splicing is not affected. We have included a figure (Supplementary Figure 4) to show that in three-intron-containing genes whose splicing is not affected in *Δsaf5* cells (ratio of splicing efficiency saf5/WT > 0.9), the splicing of the subsequent introns is also not affected.

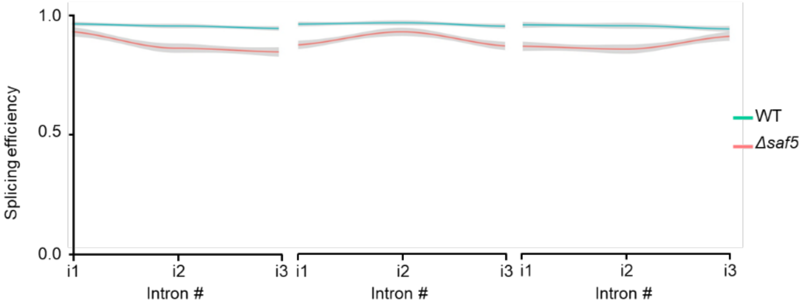

11) Lines 253-254: "The addition of the bulky ATP analog barely affected intron retention (Figure 5A). However, % of intron retention is decreased about 2-fold (I would argue that this is a significant change) after 10´of Cdk9 inactivation in the Cdk-as strain. (Wouldn't a control with the wild type strain treated with 3-MB-PP1 be necessary here?) Furthermore, the effect of Cdk9 inactivation on % of intron retention in the saf5 ko strain is also about 2-fold. It could therefore be argued that Cdk9 inactivation has a similar fold effect in improving intron splicing in the presence or absence of saf5. The same argument applies to the results of Figure 5B. Could Cdk9 inactivation attenuate the splicing defects caused by the perturbation of any other splicing factor (e.g. by providing a wider time window for the spliceosome to assemble / be activated?), i.e. is the link between saf5-dependent splicing and transcription specific of saf5?

Thanks for your suggestion. We have repeated these experiments and has also included the wild type strain treated with 3-MB-PP1, which was missing in our previous figure. All these experiments have been repeated and we have replaced the previous Figure 5.

We show that only the *cdk9-as Δsaf5* improves the splicing of *cbf11* i1, *atg5* i1 to i3, and *urm1* i1 and i3 with statistical significance. We agree with the reviewer that the % of intron retention improves after the treatment in the cdk9-as strain, but also improves splicing in the WT strain (dark orange bars). This is probably due to a side effect of the 3MB-PP1, and it is not comparable to the improvement that we observe in the cdk9-as Δsaf5 strain. Indeed, we cannot preclude any other effect (independent of Saf5) on splicing when transcription is slowed down.

We would also like to mention that following a suggestion from another reviewer we have now added a new experiment in which we have slowed transcription by treatment with Actinomycin D (which slows transcription independently of RNA Polymerase CDT phosphorylation), and we have obtained the same results: in

the absence of saf5, the splicing of the introns that we have tested, show a significant improvement (dark blue bars) (Supplementary Figure 5).

12) The Discussion is succinct and could be enriched by discussing models by which RNA polymerase elongation rates can influence the effects of saf5 depletion over other defects in RNA processing; further discussion of known functions of saf5 would be also helpful for the general reader.

We have extended the Discussion of our manuscript, following the suggestion of the reviewers, including 2 paragraphs discussing the possible role of Saf5 in regulation of splicing and the link between splicing and transcription:

> *Saf5 is conserved from yeast (LOT5 in S. cerevisiae) to metazoans and the human ICLN gene (CLNS1A) complements the Δsaf5 slow-growth phenotype [32]. ICLN, together with WD45 and the PRMT5 methyltransferase forms a complex termed methylosome that acts together with the SMN complex during the early steps of snRNP biogenesis [38]. The core spliceosome consists of five snRNPs and the biogenesis of these snRNPs is an ordered multistep process where ICLN acts as assembly chaperone assisting in the recruitment and the formation of the Sm core protein complex on the snRNA [39]. Although these functions have not yet been rigorously confirmed for Saf5 in S. pombe, the functional complementation by the human homolog points towards a conserved role.*
>
> *Research spanning several decades has provided substantial evidence indicating that a significant portion of splicing occurs co-transcriptionally. Transcriptional dynamics can influence the selection of splice sites within pre-mRNA, thereby leading to alternative splicing events and the generation of diverse mRNA isoforms [40-43]. The functional interplay between transcription and splicing is proposed to be governed by spatial and kinetic mechanisms [44]. Central to both mechanisms is the carboxy-terminal domain (CTD) of RNA Polymerase II (Pol II), whose phosphorylation status regulates transcription elongation rates and facilitates physical interactions with splicing factors such as Prp40, thereby facilitating association with the U1 complex [45]. The kinetic model posits that differential Pol II elongation rates may dictate the availability of splice sites [42, 44]. The data presented herein unequivocally establish a correlation between highly transcribed genes and the indispensable role of Saf5 in ensuring efficient splicing. Given its functional homology to ICLN, it is tempting to speculate that the absence of Saf5 creates a bottleneck in the biogenesis of functional snRNPs. The functional coupling of rapid transcription rates with splicing may place a heightened demand on snRNPs, with highly transcribed genes relying particularly on Saf5 for adequate snRNP supply. In scenarios where snRNP levels are insufficient, splicing and transcription may become uncoupled and fail to match the pace of transcript production. This potentially elucidates why the splicing of highly transcribed genes exhibits heightened dependence on Saf5. Future investigations will be required to validate this hypothesis and deepen our understanding of the*

intricate relationship between transcription, splicing, and snRNP biogenesis.

Reviewer #3: The article "*A systematic screen identifies Saf5 as a link between splicing and transcription in fission yeast*" disease by Sonia Borao et al provides genetic evidence that Saf5 mutant yeast strains have a splicing deficit. Subsequently, the authors posit the idea that the splicing defect seen in the Saf5 mutants represents a link to splicing and transcription because the splicing defect is more pronounced in transcripts that are highly transcribed. The conclusions of some of the experiments are not well supported by the data and the claims seem premature, more like the beginning of an interesting story. The discussion is brief and superficial and could use some thoughtful consideration as a way to convince the readers that these findings are of substantial interest to the splicing field.

We would like to thank the reviewer for his/her insightful comments. As she/he will discern, we have diligently endeavored to incorporate all of their recommendations, which, in our estimation, have significantly enhanced the manuscript.

Major Points.

When describing Figure 1C, the authors state that the partial inactivation of Prp2 affected all of the constructs except the one lacking an intron between the fluorescent markers (cDNA). Is this true? There seems to be very little effect on the WT or construct with the branchpoint mutant. These data should be carefully interpreted and the text updated to reflect the data if necessary.

As shown in the Figure, partial inactivation (grey bars) reduced to some extent the YFP/RFP ratio in all the constructs. We agree with the reviewer that the effect on 5'SS mut and dmut is quite obvious, and the effect on the WT and BP mut reporter in subtle, but no doubt that there is some effect. We have changed the text following the reviewer recommendation:

> *As shown in Figure 1C, the partial inactivation of Prp2 affected all the constructs except the one lacking an intron between the two fluorescent markers (cDNA). The constructs containing the wild type intron or a mutation in the BP showed a slightly but consistently reduced YFP/RFP ratio. The most pronounced impact was observed in those constructs in which the last nucleotide preceding the 5'SS was mutated (G-to-U, 5'SS mut reporter). This effect was further increased when the BP was also mutated (dmut reporter).*

For Supporting Figure 1, an n of one is shown and there is not quantification of splice ratios.

This is an experiment that was done several times. Here, we are showing three of the repeats (for the reviewer). We have added the quantification of the splice ratios to the figure, which is now included in Figure 2B.

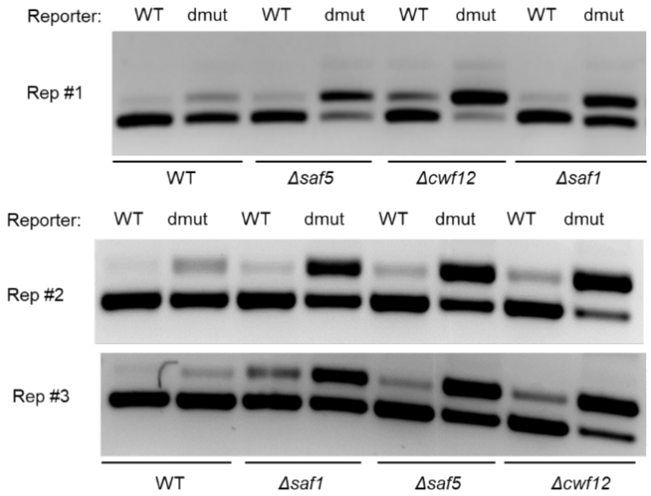

Referencing Figure 2B, the authors state that “Notably, the combination of the two mutations did not result in a simple additive effect, as the predominant splicing defects were primarily caused by the 5'ss mutation.” I agree that the splicing defects were primarily the result of the 5’ss mutation, but also do see an additive effect. Please revisit and edit the language if necessary.

Thanks. We have changed the sentence following the reviewer recommendation: *“The synergy between the two mutations (5’ss mut and BP mut) led to an additive effect, with the primary splicing defects predominantly attributed to the 5'ss mutation.”*

The authors state that cwf12 had a modest impact on splicing, but it not clear what modest means. IS the data significant? Similarly, the authors state t *On the contrary, when saf5 was deleted we observed a substantial impact on splicing* but statistical analyses are not reported for this data either.

Supporting Fig 2A compares the splicing efficiency in the mutant strains *prp2*-1, *Δsaf5* and *Δcwf12* with the splicing efficiency in a wild-type strain (or at permissive temperature for the *prp2-1* strain). The impact on splicing is higher in a *Δsaf5* than in a *Δcwf12* strain, as in can be seen by the tendency lines (blue lines) which are the result of a polynomic regression. The formula for the tendency lines for each strain is:

prp2-1: $y = -0.892 + 0.549e^x$; $R^2 = 0.26$
Δsaf5: $y = -0.71 + 0.585e^x$; $R^2 = 0.61$
Δcwf12: $y = -0.526 + 0.537e^x$; $R^2 = 0.77$

We have added this information in the figure legend. This tendency line indicates how far is the splicing efficiency of a given strain from the wild type strain for each single intron. In the case of the *prp2*-1 strain (it is worth to remember that *prp2* is an essential gene), when placed at 34ºC the cells stop growing, so it makes sense that the tendency line (blue) is so different from a ratio of splicing 1:1 (black line); in the case of *Δcwf12* the tendency line is very similar to a 1:1 ratio (blue and black lines are very similar); finally, in the case of *Δsaf5* we interpret that its tendency line is very different from what we obtained for *Δcwf12* (although is not so extreme as the one obtained for *prp2-1*).

Also, as shown in Figure 3A, the effect on splicing was more profound in *Δsaf5* cells that in *Δcwf12* cells, which is especially notorious in the 6 to 10 percentiles, where the differences between both strains are more marked. Even though, as mentioned above, the differences of both strains (*Δsaf5* and *Δcwf12*) with the wild type strain are statistically significant in all the percentiles.

Is the data in Figure 3 statistically significant?

We have now calculated the statistical significance in Figure 3A, and included the following sentence in the figure legend: *"Significant differences were calculated using a two-sided t-test comparing the values of splicing efficiency of each decile in Δsaf5 or Δcwf12 strains with the corresponding decile in the wild type strain. All comparisons were statistically significant (p<0.001)."*

When referring to the mmi1 gene, a 4 intron gene that has suboptimal splicing of its introns, the authors stat that the *cumulative effect on the final mRNA would result in only 44% of mature mRNA with the correct splicing in all four introns.* It is not clear how the authors came up with this number. The 44% number seems to assume that splicing efficiency of individual introns is not affected by the splicing efficiency of other introns in the same transcript, which may be true, but is not necessarily true.

Yes, the reviewer is right: we have assumed that the effect on *mmi1* splicing comes from the added effect of the splicing of the individual introns (74%, 71%, 95% and 85%, respectively). It is noteworthy to indicate that fission yeast has no mayor reported alternative splicing events (most of the regulatory effects on splicing belong to the category "intron retention"). Furthermore, we have previously shown (Hummer et al, Cell Reports, 2021) that genes with 2 introns in which one is close to 100% efficiently spliced and the other close to 100% retained, this is unaffected by the order in which the introns are placed.

Furthermore, we can rule out some kind of crosstalk between the splicing of different introns of the same pre-mRNA in the case of *mmi1*: the theoretical splicing efficiency that we calculated for *mmi1* correlates quite well with what we have observed *in vivo*: the amount of Mmi1 in *Δsaf5* is reduced to approx. 45%, when compared to the wild type strain (as shown in new Supporting Figure 3B):

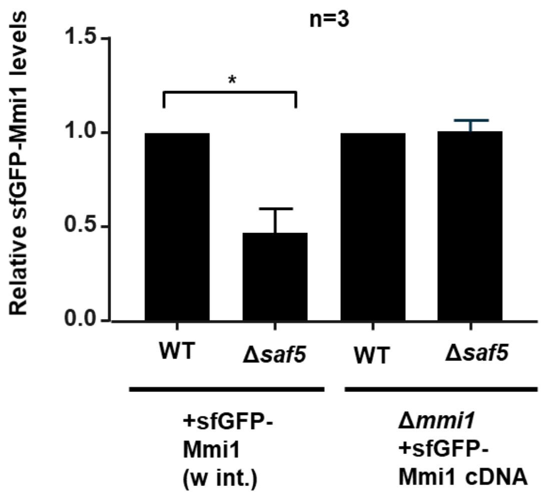

The data in Supporting Figure 3 A is not clear and appears to be from an n of one. This data should be repeated, analyzed, and supported with statistical analysis.

This experiment was done several times, but we only showed 1 repeat. Here we show the other repeats of the experiment shown in Supporting Figure 3A, as requested by the reviewer. Statistical analysis has been included in the Supporting Figure 3B and the graph was shown in the previous answer to the reviewer.

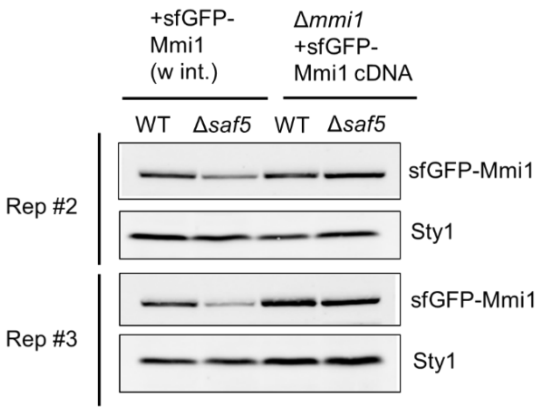

The data inf Supporting Figure 3B is not conclusive. An appropriate control for this experiment would be the +sfGFP-Mmi1 with introns to compare directly to the +Mmi1 cDNA. It would also be nice if expression could be validated.

Before expressing non-tagged Mmi1 cDNA, we performed several attempts to compare the effects on the expression of some meiosis genes (*rec10*, *bqt1*) in strains expressing sfGFP-Mmi1 or Mmi1-sfGFP, with and without introns. However, when we analyzed the levels of the mRNA for the sfGFP-tagged (both in the N- or C-terminal) *mmi1*, we realized that the expression levels were much higher than the endogenous *mmi1*, suggesting that the sfGFP was stabilizing the mRNA of the chimeric genes. To circumvent this problem (and to add only an extra copy of Mmi1) we had to express the gene without tags (old Supporting Figure 3B). As we show in the figure (now in the Supporting Figure 3D), both WT and *Δsaf5* cells express only one extra copy of *mmi1* in addition to the endogenous one when the constructs containing *mm1* (cDNA or with introns) do not contain sfGFP.

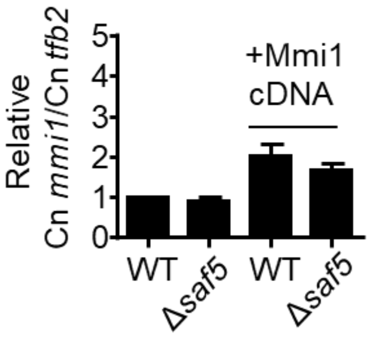

Figure 4A. It is not clear that coldshock has an effect on growth that is communicated in the text. Please add replicates, quantification, or both to substantiate this claim.

We say in the text (p. 10) that *Δsaf5* cells "were temperature sensitive and cold sensitive"; this is in agreement with what was previously published in Barbarossa et al (2014) (ref. 32 in the manuscript), where they indicate that *Δsaf5 "displayed a noticeable growth defect, since they form small colonies with a growth rate more than 2-fold lower than that of the wild type when grown at 25°C, and they are temperature sensitive at 37°C (Fig. 1C)."* We have included this reference when we mention the phenotype that we observed in the *Δsaf5* cells.

Regarding the replicates, we have done this spot assay several times (see image for the reviewer below with 3 replicates). We do not understand what exactly means the reviewer with the quantification of a spot assay, since we always have considered this kind of assay as a qualitative assay, not a qualitative assay.

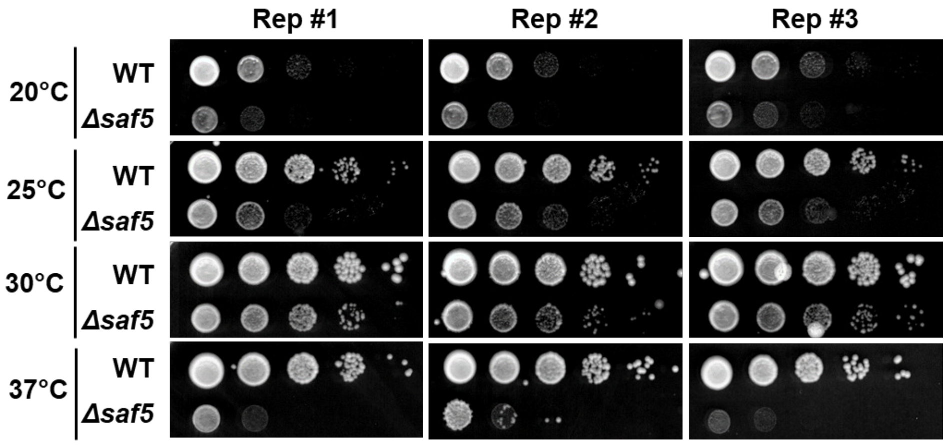

Referring to Figure 4B, the authors state that only relevant categories are shown. How is the "relevance" determined?

Since GO term are redundant, we have chosen only one of the terms that represent related GOs. We have indicated this in the Figure Legend.

Figure 4E shows *** to denote statistical significance between the 2 groups shown, but the meaning of *** is not disclosed.

Sorry, our mistake. We have now included the information in the figure legend (*** $p<0.001$)

Figure 5A seems to either be lacking statistical analysis, or to not have statistically significant data. Please clarify and modify the text accordingly.

We have changed the entire figure: we have also included the WT strain and included the statistical analysis in all the graphs of the figure (which were missing in the previous version). We have changed the Figure Legend accordingly.

The effect on the rate of transcription should be quantified for Figure 5.

We have repeated exactly what was published by the Fisher lab some years ago (references 34 and 35 in our manuscript). In fact, we obtained the strains and the protocol to inactivate Cdk9 from them. In those manuscripts (and in several more published later) it is clearly described that inhibition of Cdk9 using an allele that can be inactivated with bulky ATP slows down transcription. Furthermore, we have also used an alternative method to slow down transcription (Actinomycin D), which replicates our results with the Cdk9_as allele (see below; new Supporting Figure 5).

In Figure 5B and 5C, what is the explanation for the cdk9-as intron retention values going down over time to mimic the response seen in the cdk9-as deltasaf5 activity (in the atg5i2 atg5 i3 and urm1 i3 samples)? This should be discussed in the manuscript.

We believe that the improvement in splicing is caused by the slowdown of transcription: if a given intron is not fully spliced (even in the presence of Saf5), slowing down transcription improves splicing. What happens is that in the absence of Saf5, splicing is worst in those introns, so there is mucho more room for improvement.

There are 2 facts that support this:

1) We have now added the values for WT; the reviewer can observe that splicing is not improved when cells are treated with 3-MB-PP1 (orange columns)

2) If we test an intron whose splicing is not affected in *Δsaf5* with an intron retention of less than 1% (see figure below), there is no such improvement effect.

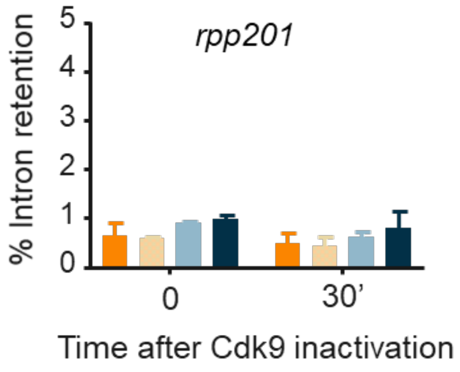

We have added a sentence in the manuscript discussing this observation: *"Splicing also improved in the cdk9as saf5+ strain (depicted by the pale orange bars), probably as the result of decreasing transcription rate and giving more time for the splicing to occur, however this improvement was not statistically significant."*

The data in figure 5 would be strengthened by a second method to slow transcription and duplicate the results.

We have now assayed an alternative method to slow transcription, using Actinomycin D. This drug affects transcription independently of RNA pol CTD phosphorylation. As now can be seen in the new figure (Supporting Figure 5), we can recapitulate the results obtained with the inhibition of Cdk9.

Minor Points.

In the abstract, please define a “life construct.”

Following the recommendation of Reviewer #1, we have changed the term “life construct” with “integrated fluorescence-based *in vivo* splicing reporter construct”, which is self-explanatory term.
