# Supplementary material for: A systematic screen identifies Saf5 as a link between splicing and transcription in fission yeast
Source: PLoS Genet. 2024 Jun 4;20(6):e1011316. doi: 10.1371/journal.pgen.1011316 (PMC11178228; doi:10.1371/journal.pgen.1011316)
Supplement: Attachment — Submitted filename: Response to Reviewer.pdf [file pgen.1011316.s013.pdf]

Reviewer #1: The authors have addressed my concerns. I support its publication in the current form.
Reviewer #4: The authors have adequately addressed the issues raised in my previous report and I recommend publication of the manuscript in PLoS Genetics.
We would like to thank the reviewers for their highly constructive comments, which have clearly improved the manuscript.

Reviewer #3: The revised article by Sonia Borao et al is greatly improved by the additional data using actinomycin to slow transcription rates as well as the expanded and thoughtful discussion section. The authors prepared a careful point-by point response to the previous reviews that is very much appreciated. Replicate experiments and statistical analysis are now clearly shown.
Thanks! Your comments and suggestions have have significantly enhanced the quality of the manuscript.

To this reviewer though, there are still residual concerns, stemming from the primary conclusion that transcription rate is affected in Figure 5 and in supporting Figure 5. In response to the Reviewer 3's request to quantify the change in rate of transcription (or to show that the transcription rate is changing at all) the authors merely state that they have "repeated exactly what was published by the Fisher Lab some years ago." This seems like a historical control (and done by another lab!!) than cannot be acceptable proof that transcription rates are changing in this experiment. Likewise, there is no new data to show that actinomycin is changing transcription rates (as has been previously reported and is expected).
Thanks for the comment. While we value the reviewer's feedback, we aim to demonstrate that these concerns may not be directly pertinent to the central aim of the manuscript.
While it is true that we have not directly demonstrated a decrease in transcription rate using either method, we have drawn upon established findings from previous studies (Arioka et al., 1998; Viladevall et al., 2009). Our experiments utilized identical strains and chemicals, readily available commercially, as those in the original reports. Additionally, our results remained consistent across two distinct approaches: inhibition of Cdk9 and inhibition of RNA polymerase with actinomycin D.
If we correctly interpret the reviewer's suggestion, validating a change in transcription as depicted in Figure 5 and Supplementary Figure 5 would necessitate run-on experiments, which exceed the scope of our manuscript. However, in an attempt to address the reviewer's concerns, we are showing here:

1- WB analyses to illustrate that the addition of an ATP analogue to the culture effectively inhibited Cdk9, resulting in the dephosphorylation of RNA polymerase II CTD Ser2. As the reviewer is aware, Ser2 phosphorylation is a marker of transcription elongation; hence, we are sure that, in our hands, inhibiting this phosphorylation event leads to the downregulation of transcription elongation.
2- The reviewer will agree with us that the effect of actinomycin D on transcription is known for almost 4 decades (H.M. Sobell, PNAS, 1985) and its use in fission yeast is widely described (reference 39 in our manuscript). We can confirm to the reviewer that cells treated in the same exact conditions as described in the manuscript stop their growth (assayed by growth curves and spot dilutions); thus, we can conclude that their transcription rate should be decreased.
3- We have tested 2 different and unrelated methods to decrease transcription and have obtained the same results: an alleviation of the effect on intron retention on highly transcribed genes.

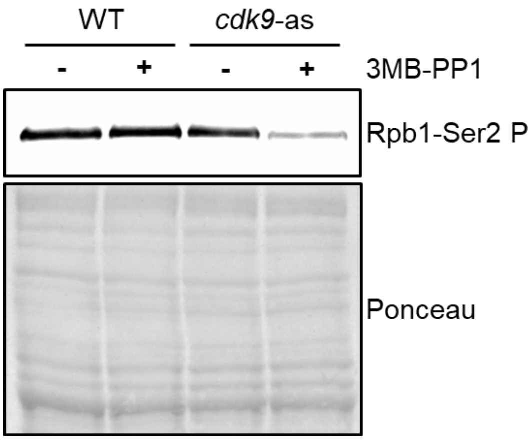

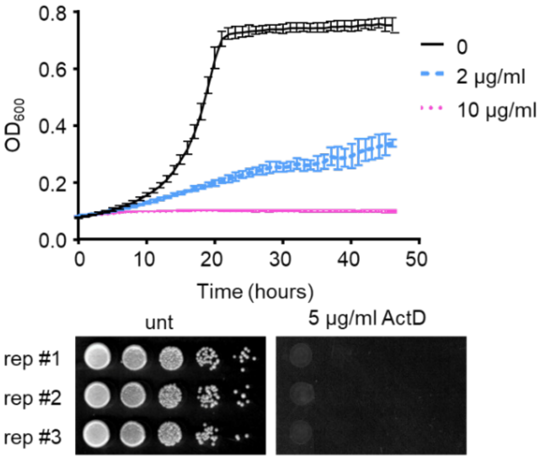

There are other examples where the data seem to be over-stated or interpreted inconsistently:

- The authors seem to incorrectly conclude that "The constructs containing the wild type intron or a mutation in the BP showed a slightly, but consistently reduced YFP/RFP ratio" in Figure 1, when in fact there is no change seen. The only changes seen are in the 5'ss and double mutant.

We want to believe that there is a problem with the image downloaded by the reviewer. For us, it is clear that those 2 constructs have SLIGHTLY reduced YFP/RFP ratio. The YFP/RFP ratio shows a decrease of 1% and 12% for the WT reporter at 25ºC or 30ºC, respectively, when compared to the cDNA ratio; and, similarly, a 5% and 8% for the BP mut reporter at 25ºC or 30ºC, respectively. Obviously, the decrease is more pronounced in the 5'SS and dmut reporters, where splicing drops up to 25% or 76%. Please, review the figure, where we have annotated the YFP/RFP ratio in red for both temperatures:

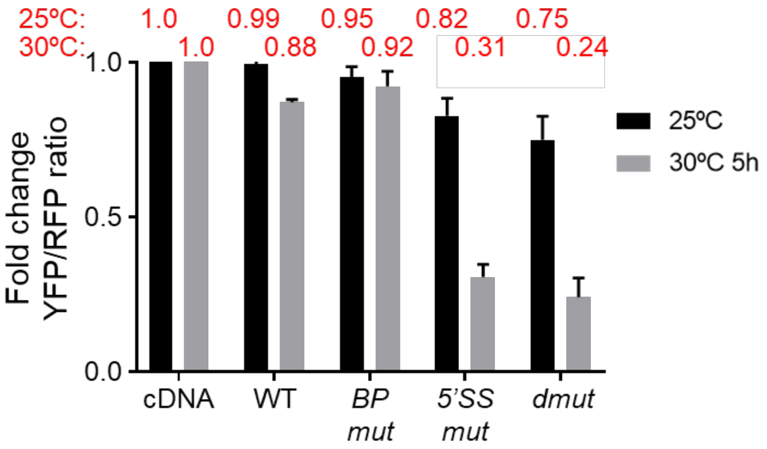

Thus, in all the reporters there is a reduction in the slicing efficiency when compared to the cDNA reporter, although is more evident in the 5'SS and dmut reporter. Even though, and to avoid confusion, we have changed the text of the manuscript, putting emphasis in the condition in which we partially inactivate Prp2 (30ºC): "The constructs containing the wild type intron or a mutation in the BP showed a slightly, but consistent reduction of the YFP/RFP ratio (12% and 8%, respectively; Figure 1C) when Prp2 was partially inactivated at 30ºC"

- The authors state that “The synergy between the two mutations (5’ss mut and BP mut) led to an additive effect, with the primary splicing defects predominantly attributed to the 5'ss mutation.” Since synergy, by definition, is a sum greater than its parts (ie not simply additive), this statement should be corrected.
Thanks. We have changed the sentence to “The combination of the two mutations (5’ss mut and BP mut) led to an additive effect, with the primary splicing defects predominantly attributed to the 5'ss mutation”

- For Figure 5A, the authors state that “Splicing also improved in the cdk9as saf5+ strain (depicted by the pale orange bars).” This reviewer is unable to discern that improvement in the figure.
The intron retention in the cdk9-as strain goes from **23%** before treatment to **10%** after 30 minutes of treatment, which is more than 50% reduction on the intron retention (that is, more than a 50% improvement). The figure shown below is extracted from Figure 5, marking the differences that are shown in the original figure. So we can say that yes, there is improvement in the splicing:

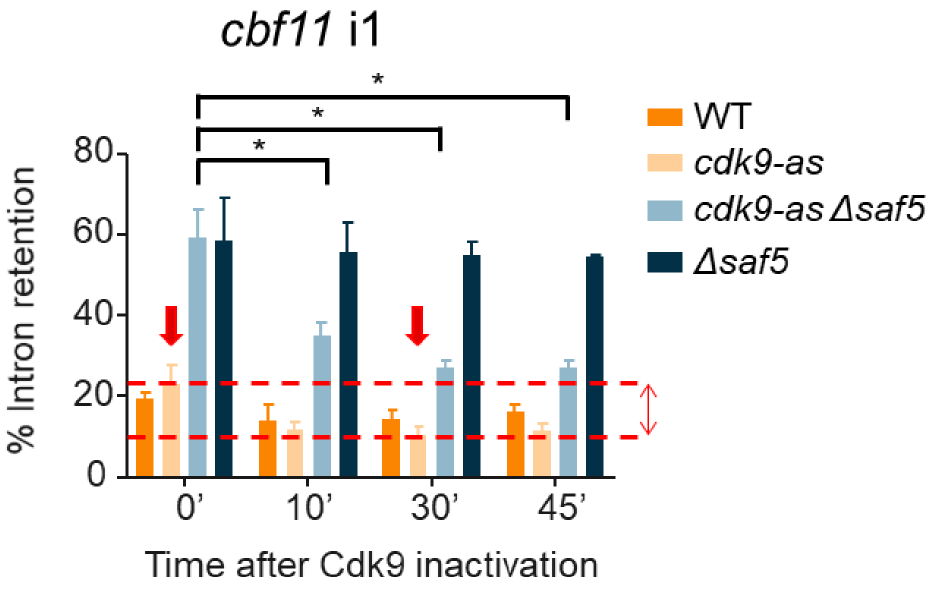

- The authors added a new Figure 5D to show that splicing is not affected in an intron in which retention is less than 1%. However, is difficult to imagine that splicing could be mor efficient than 99% so this point does not seem to support the argument.
From our RNAseq experiments, we have detected **221 introns** whose splicing is 100% in a wild type strain and **659 introns** with a splicing efficiency between 99% and that do not reach 100%. This means that there is a total of **880 introns** whose splicing is better than 99%. So, we can argue that in fact, yes, there is room for improvement, even when starting with an excellent 99% of splicing.

Other points.

Does the author mean “degenerate” when stating “degenerated” on Page 14?
Thanks, we have corrected the typo

In the following sentence “As shown, we could not observe any effect on splicing in neither case”, neither should be replaced by either.
Thanks, we have corrected the typo
