# Supplementary material for: A systematic screen identifies Saf5 as a link between splicing and transcription in fission yeast
Source: PLoS Genet. 2024 Jun 4;20(6):e1011316. doi: 10.1371/journal.pgen.1011316 (PMC11178228; doi:10.1371/journal.pgen.1011316)
Supplement: Attachment — Submitted filename: Borao et al review 2024.docx [file pgen.1011316.s011.docx]

The article “*A systematic screen identifies Saf5 as a link between splicing and transcription in fission yeast*” disease by Sonia Borao et al provides genetic evidence that Saf5 mutant yeast strains have a splicing deficit. Subsequently, the authors posit the idea that the splicing defect seen in the Saf5 mutants represents a link to splicing and transcription because the splicing defect is more pronounced in transcripts that are highly transcribed. The conclusions of some of the experiments are not well supported by the data and the claims seem premature, more like the beginning of an interesting story. The discussion is brief and superficial and could use some thoughtful consideration as a way to convince the readers that these findings are of substantial interest to the splicing field.

Major Points.

When describing Figure 1C, the authors state that the partial inactivation of Prp2 affected all of the constructs except the one lacking an intron between the fluorescent markers (cDNA). Is this true? There seems to be very little effect on the WT or construct with the branchpoint mutant. These data should be carefully interpreted and the text updated to reflect the data if necessary.

Foe Supporting Figure 1, an n of one is shown and there is not quantification of splice ratios.

Referencing Figure 2B, the authors state that “Notably, the combination of the two mutations did not result in a simple additive effect, as the predominant splicing defects were primarily caused by the 5'ss mutation.” I agree that the splicing defects were primarily the result of the 5’ss mutation, but also do see an additive effect. Please revisit and edit the language if necessary.

The authors state that cwf12 had a modest impact on splicing, but it not clear what modest means. IS the data significant? Similarly, the authors state t *On the contrary, when saf5 was deleted we observed a substantial impact on splicing* but statistical analyses are not reported for this data either.

Is the data in Figure 3 statistically significant?

When referring to the mmi1 gene, a 4 intron gene that has suboptimal splicing of its introns, the authors stat that the *cumulative effect on the final mRNA would result in only 44% of mature mRNA with*

*the correct splicing in all four introns.* It is not clear how the authors came up with this number. The 44% number seems to assume that splicing efficiency of individual introns is not affected by the splicing efficiency of other introns in the same transcript, which may be true, but is not necessarily true.

The data in Supporting Figure 3 A is not clear and appears to be from an n of one. This data should be repeated, analyzed, and supported with statistical analysis.

The data inf Supporting Figure 3B is not conclusive. An appropriate control for this experiment would be the +sfGFP-Mmi1 with introns to compare directly to the +Mmi1 cDNA. It would also be nice if expression could be validated.

Figure 4A. It is not clear that coldshock has an effect on growth that is communicated in the text. Please add replicates, quantification, or both to substantiate this claim.

Referring to Figure 4B, the authors state that only relevant categories are shown. How is the “relevance” determined?

Figure 4E shows *** to denote statistical significance between the 2 groups shown, but the meaning of *** is not disclosed.

Figure 5A seems to either be lacking statistical analysis, or to not have statistically significant data. Please clarify and modify the text accordingly.

The effect on the rate of transcription should be quantified for Figure 5.

In Figure 5B and 5C, what is the explanation for the cdk9-as intron retention values going down over time to mimic the response seen in the cdk9-as deltasaf5 activity (in the atg5i2 atg5 i3 and urm1 i3 samples)? This

should be discussed in the manuscript.

The data in figure 5 would be strengthened by a second method to slow transcription and duplicate the results.

Minor Points.

In the abstract, please define a “life construct.”
